# Chemistry and Functionality of Health-Promoting Bioactive Compounds: An Overview

**DOI:** 10.3390/molecules31122179

**Published:** 2026-06-22

**Authors:** Elena Enachi, Iuliana Aprodu, Leontina Grigore-Gurgu

**Affiliations:** 1 Faculty of Food Science and Engineering, Dunărea de Jos University of Galati, 111 Domnească Street, 800201 Galați, Romania; 2 Faculty of Medicine and Pharmacy, Dunărea de Jos University of Galati, 47 Domnească Street, 800008 Galați, Romania

Bioactive compounds from natural resources play an important role in promoting human health due to their ability to lower the oxidative stress, contribute to the prevention of chronic diseases, and influence various physiological processes. Through complex mechanisms of action, these compounds intervene in the regulation of the inflammatory response, protect cellular structures against oxidative damage, and support the normal functioning of the metabolic, immune, and cardiovascular systems, thus having a significant impact on quality of life and longevity. This Special Issue of Molecules, entitled “Food Chemistry and Bioactive Compounds in Relation to Health—Second Edition”, brings together six original research articles and three review papers that target advanced aspects related to the compositional characterization, modern methods of extraction, and valorization of bioactive compounds, as well as the functional evaluation of their biological activities, including antioxidant, antimicrobial, and cytotoxic properties and applications in the food, pharmaceutical, and biotechnological fields.

The studies included in this Special Issue focus on scientific subjects, which can be grouped into several categories: (i) the role of phenolic compounds in the prevention and management of chronic diseases, especially chronic kidney disease, highlighting the antioxidant and anti-inflammatory mechanisms mediated by Nrf2 pathway activation and NF-κB inhibition, as well as the correlations between diets rich in polyphenols and slowing the decline of renal function; (ii) the characterization of the inorganic composition of processed protein products and the use of mineral profiling for the authentication and differentiation of the source of origin, including highlighting the high nutritional potential of insect-derived proteins; (iii) the valorization of agri-food by-products rich in bioactive compounds, such as those from the wine and tomato industries, by analyzing the phenolic composition as well as antioxidant and antimicrobial activities and identifying applications in the food, cosmetic, and pharmaceutical fields; (iv) the antioxidant and antimicrobial properties of aromatic plants from the *Lamiaceae* family, correlated with the content of phenolic and terpenic compounds, as well as the optimization of modern extraction methods to increase the yield and stability of bioactive compounds; (v) obtaining and characterizing innovative systems with biomedical and food potential, including synthetic coumarin derivatives with cytotoxic and antioxidant activity, as well as nanoformulations based on vitamin D_3_ and silver nanoparticles with improved antibacterial properties; and (vi) developing functional systems for the delivery and stabilization of bioactive compounds, such as protein aggregates for the encapsulation of polyphenols, and the study of the fungal secretome as a source of hydrolytic enzymes with biotechnological applications. To facilitate the exploration of these contributions, a brief integrative description of each study is provided in the following sections.

In their systematic review, Baptista et al. [Contribution 1] analyzed the role of phenolic compounds in the prevention, adjuvant treatment, and management of chronic kidney disease (CKD). The authors evaluated the evidence from 71 preclinical and clinical studies investigating various classes of polyphenols, including flavonoids (catechins, quercetin, kaempferol), phenolic acids (caffeic, ferulic, gallic acid), stilbenes (resveratrol), lignans, and tannins, in the context of oxidative stress, chronic inflammation, and progression of renal dysfunction. Most of the targeted compounds demonstrated antioxidant and anti-inflammatory activity, with renoprotective effects mediated mainly by activating the Nrf2 (nuclear factor erythroid 2-related factor 2) pathway and inhibiting the NF-κB (nuclear factor kappa light -chain enhancer of activated B cells) pathway, central mechanisms involved in the pathogenesis of CKD. In multiple experimental models of induced nephropathy (diabetic, drug-induced, or toxic), polyphenols caused a reduction in biochemical markers of renal damage (urea, creatinine, BUN, albuminuria), simultaneously improving histopathological lesions, such as renal fibrosis and tubular atrophy. Observational and interventional studies also suggested that diets rich in polyphenols are associated with a slower progression of renal function decline and a reduction in cardiovascular risk in patients with CKD. Their results are consistent with those reported by Casanova et al. [1], who stated that dietary antioxidants, especially polyphenols, may contribute to slowing the progression of CKD by reducing the oxidative stress and systemic inflammation, although clinical efficacy depends on the type of compound and the stage of the disease. The concluding remarks of the review re also supported by the data presented by Choi et al. [2], who demonstrated that a diet centered on plant-based foods, rich in polyphenols, is associated with a significantly lower risk of renal function deterioration in the early stages of chronic kidney disease.

Inaudi et al. [Contribution 2] investigated the inorganic composition of processed animal protein (PAP) feeds to evaluate the potential of using the mineral profile as a tool for the discrimination of species of origin and the authentication of feeds. PAPs from bovine, porcine, poultry, and fish sources were analyzed, as well as PAPs from feathers, plant feeds, and three types of insects (*Acheta domesticus*, *Bombyx mori*, *Tenebrio molitor*). The determinations of the major and trace elements was performed using ICP-OES and of inorganic anions via ion chromatography (IC), and the data were subsequently interpreted using PCA-type chemometric analysis. The results revealed clear quantitative differences between the PAP categories. Fish PAPs showed significantly higher concentrations of Cd (~1.20 mg/kg), Sr (~228 mg/kg), Na (~9.06 g/kg) and Mg (~2.37 g/kg), reflecting the bioaccumulation tendency specific to aquatic organisms. In contrast, porcine PAPs were characterized by a lower overall content of metals, for example, Fe ~54 mg/kg and Zn ~43 mg/kg, a phenomenon attributed to the higher proportion of adipose tissue. Poultry and cattle PAPs showed high levels of structural macroelements, especially Ca (~100 g/kg and 49–57 g/kg) and P (~58 g/kg and 27–32 g/kg). Insect PAPs were distinguished by very high contents of Zn (up to ~211 mg/kg in *Acheta domesticus*), Cu (9–27 mg/kg), and K (8.75–11.1 g/kg), values comparable to or higher than those found in vertebrate PAPs, indicating a higher nutritional potential. In addition, feather PAPs and vegetal feed showed similar profiles for Al (110–118 mg/kg), Si (266–271 mg/kg) and Ni (1–1.15 mg/kg), but feathers were differentiated by higher concentrations of Fe (~279 mg/kg) and Zn (~120 mg/kg). The PCA confirmed that elements such as Cd, Sr, Zn, and Cu contribute significantly to the differentiation of the species of origin. These results agree with those reported by Pederiva et al. [3], who found that insect-derived PAPs presented high concentrations of zinc and copper, supporting the use of insects as sustainable alternative sources of micronutrients in animal nutrition. The elevated levels of cadmium and strontium identified in fish PAPs were similar to those observed by Franco-Fuentes et al. [4], who described the bioaccumulation of these elements in marine organisms and their importance in toxicological risk assessment.

Silva et al. [Contribution 3] assessed the phenolic composition, antioxidant activity, and antimicrobial potential of winemaking by-products, seeds, skins, and bunches from three red grape varieties, namely, Periquita, Gamay, and Donzelinho Tinto, from the Douro wine region. Phenolic compounds were analyzed using HPLC-DAD, and 11 phenolic compounds were identified and quantified, including phenolic acids, flavan-3-ols, flavonols and anthocyanins. The results revealed significant quantitative differences between grape parts. The seeds were characterized by high flavan-3-ol contents, especially catechin and epicatechin, with a total flavan-3-ol content of 12.92 mg/g dry matter in Gamay, compared to 6.77 mg/g in Periquita seeds. Epicatechin gallate was detected only in trace amounts. The skins had the highest total polyphenol content, dominated by the anthocyanin malvidin-3-O-glucoside, with values ranging from 39.1 to 42.6 mg/g dry matter, depending on the variety. The stems were the only fractions that contained the hydroxycinnamic acids caftaric and coutaric, with a total content of up to 1.49 mg/g for the Periquita variety. The antioxidant activity, assessed using the DPPH assay, showed that the seed extracts had the highest free radical scavenging capacity, with IC_50_ values of 0.23 mg/mL for Gamay seeds, 0.36 mg/mL for Donzelinho Tinto, and 0.43 mg/mL for Periquita, whereas, for the skins of the three varieties, the values were 0.90, 0.98, and 0.72 mg/mL, respectively. The seed extracts also demonstrated concentration-dependent activity against •NO and O_2_•^−^, with IC_25_ values up to 0.38 mg/mL for the superoxide radical, confirming the superior antioxidant potential of this fraction. Regarding antimicrobial activity, the extracts showed a more pronounced effect on Gram-positive bacteria, especially *Listeria monocytogenes*, with minimum inhibitory concentrations (MICs) of 10–25 µg/mL for the seed extracts. The activity against Gram-negative bacteria (*Escherichia coli*) was limited, being observed only in a few strains isolated from pig farms, suggesting higher resistance is associated with the structure of the bacterial cell wall. These results are in line with those reported by Silva et al. [5], who found that grape seed extracts exhibit superior antibacterial activity compared to other winemaking by-products, especially against Gram-positive bacteria. The high levels of malvidin-3-O-glucoside in the skin confirmed the observations of He et al. [6], who indicated that this anthocyanin was the main phenolic pigment responsible for the antioxidant capacity of red grapes and wines.

Grigore-Gurgu et al. [Contribution 4], in their review, investigated the antioxidant and antimicrobial potential of aromatic plants from the *Lamiaceae* family (basil, marjoram, oregano, rosemary, sage, thyme, and summer thyme), highlighting the major bioactive compounds, extraction methods, and fields of applicability. The authors showed that these plants are important sources of phenolic acids (rosmarinic, caffeic), flavonoids, phenolic diterpenes (carnosic acid, carnosol), and terpenic volatile compounds (thymol, carvacrol, linalool), which have high antioxidant capacity and significant antimicrobial activity. The total polyphenol contents varied widely amongst the plants, from approximately 1815 mg/100 g d.w. in thyme to over 4512 mg/100 g d.w. in summer savory, with the values being influenced by the species, cultivation conditions, and extraction method. The antioxidant activity reported by the authors closely correlated with the concentration of phenolic compounds, a relationship also confirmed by comparative analysis with the Phenol-Explorer database, previously reported by Neveu et al. [7], which indicated rosmarinic acid as the main antioxidant contributor in most of the *Lamiaceae* species. Regarding their antimicrobial activity, thyme and oregano essential oils stand out for their efficiency against Gram-positive bacteria and multidrug-resistant strains, results consistent with the experimental data presented by Ilić et al. [8], who attributed the strong bactericidal effect to the presence of thymol and carvacrol. In addition, the authors emphasized the advantages of modern extraction methods such as ultrasound, microwave, supercritical fluid methods, which ensure higher yields and the stability of bioactive compounds, supporting the use of aromatic plants as natural alternatives to synthetic antioxidants and antimicrobials, with extensive applications in the food, pharmaceutical, cosmetic, active packaging, and biopesticides industries.

Petraglia et al. [Contribution 5] performed a comparative analysis of the enzyme profile of the secretome of *Pleurotus eryngii* and *Pleurotus ostreatus* mushrooms, cultivated on the same substrate, with the aim of evaluating the dynamics of hydrolytic enzyme secretion at three distinct developmental stages (23, 34, and 50 days). The results showed that both species secreted a broad spectrum of extracellular hydrolytic enzymes, including proteases, lipases, amylases, α-glucosidase, cellulases, and hemicellulases. *P. eryngii* consistently showed superior enzymatic activities compared to *P. ostreatus*. The total protease activity increased significantly until the complete colonization of the substrate, which remained high during the fruiting body formation phase, reaching values of approximately 774 U/g after 50 days for *P. eryngii*, a result similar to the previous observations of proteases in the *Pleurotus* genus having multiple roles, not only nutritional but also regulating other enzyme systems, as demonstrated by Inácio et al. [9]. The temporal analysis of carbohydratez-hydrolytic enzymes revealed the predominant use of the starch in the early stages, followed by an intensification of cellulolytic and hemicellulolytic activities upon complete colonization of the substrate, a pattern well-known for enzymatic secretion. In addition, the identified lipases showed a clear preference for long-chain substrates (C10–C16), confirming the presence of true lipases, with an enzymatic behavior similar to that described by Piscitelli et al. [10] for extracellular lipases from *P. ostreatus*. Overall, the study demonstrated that the secretome of both *Pleurotus* species, especially *P. eryngii*, represents a valuable source of hydrolytic enzymes with the strong potential for biotechnological applications, supporting the sustainable valorization of residual substrates from mushroom cultivation in the context of a circular bioeconomy.

Szwaczko et al. [Contribution 6] studied the effect of introducing a phosphonate group into the coumarin skeleton on the cytotoxic, antioxidant, and pharmacokinetic properties of some synthetic derivatives, tested on the colorectal cancer line HT-29 and on normal colonic epithelial cells CCD 841 CoTr. The authors synthesized a phosphonated coumarin at the C-3 position (CM-2) and two cyclic phosphocoumarins (CM-3 and CM-4) via the Knoevenagel reaction, obtaining high yields (between 69 and 81%). The in silico analysis revealed optimal values of topological polar surface area (62–75 Å^2^) and logP (2.1–3.2), all derivatives respecting the Lipinski, Ghose, Veber, Egan, and Muegge criteria for drug-likeness and oral bioavailability rules. Biologically, the phosphonate compounds induced a decrease in the mitochondrial activity of the HT-29 cells by up to 34–35% at a concentration of 200 µg/mL for CM-2 and CM-3, while CM-4 caused a reduction in the viability of approximately 45% at the same concentration according to the NR assay. The cell cycle analysis revealed an increased accumulation of cells in the sub-G1 phase and a block of the G1/S phases. The mechanism of action is similar to that reported by Khosravifar et al. [11], who showed that synthetic coumarin derivatives induce G1/S arrest as the main antiproliferative effect. From an antioxidant point of view, CM-2 showed the highest activity, having a DPPH radical scavenging capacity of ~25 µg/mL Trolox at 200 µg/mL as well as a FRAP activity comparable to ~9 µg/mL ascorbic acid. The results agree with the data reported by Stefani et al. [12], emphasizing the role of substituents in increasing the antioxidant potential of coumarin derivatives. The phosphonate functionalization of coumarin led to compounds with improved pharmacological and redox profiles as promising precursors for further development in the field of antitumor agents.

Bakirova et al. [Contribution 7] developed a supramolecular assembly of vitamin D_3_ (cholecalciferol), encapsulated in a β-cyclodextrin (β-CD) shell and functionalized with silver nanoparticles (AgNPs), with the aim of improving the solubility, stability, and biological functionality of vitamin D in food and biomedical applications. The VD_3_-β-CD-AgNP nanocomposite was obtained in an optimal molar ratio of 15:1:1 and characterization by UV–Vis, SEM, TEM, and XRD. The authors reported the formation of well-dispersed spherical nanoparticles, with dimensions ranging from 6 to 20 nm and an average diameter of 9.25 ± 1.17 nm, values comparable to those previously reported by Zargar et al. [13] for β-CD–AgNP systems intended for biological applications. Thermogravimetric analysis revealed a multi-step degradation process, in which the VD_3_–β-CD–AgNP complex presented mass losses of ~7.6% below 130 °C because of water removal and a major decomposition between 240 and 420 °C, indicating a different thermal stability compared to that of β-CD–Ag complexes without vitamin D. These observations are in agreement with the results reported by Soares et al. [14] for similar molecular complexes with vitamin D_3_. Biologically, the nanocomposite demonstrated significant antibacterial activity, with a maximum effect at a concentration of 50 µg/mL, reducing the growth rate of *Escherichia coli* by approximately 4.0 ± 0.3 times and that of the Gram-positive bacteria *Staphylococcus aureus* and *Enterococcus hirae* by 6.0 ± 0.5 and 6.5 ± 0.2 times, respectively. In addition, the number of colony-forming units (CFUs) decreased by ~48% for *E. coli*, ~60% for *S. aureus*, and ~67% for *E. hirae*, confirming the bactericidal effect of the nanocomposite. The inclusion of vitamin D_3_ in a β-CD–AgNP system targeted a stable nanostructured material with superior antibacterial properties and a high potential for food fortification and the development of functional value-added systems.

Maciejczyk et al. [Contribution 8] examined tomato pomace (TP), a by-product resulting from industrial tomato processing, as a sustainable source of bioactive compounds for cosmetic and pharmaceutical applications, with a focus on the impact of separating the peel and seed fractions for technological valorization. Tomato pomace includes 3–5% fresh tomato mass and is the result of the global processing of over 25% of the total annual tomatoes produced, which is approximately 180 million tons worldwide, with seeds representing 34–45% of the TP dry weight. From a compositional point of view, the authors showed that the peel fraction concentrated 80–90% of the total carotenoids, with a lycopene content ranging from 3.67 to 193 mg/100 g d.w., values comparable to those reported by Nour et al. [15], who highlighted the preferential accumulation of lycopene in peel-rich processing waste. In contrast, the seeds were rich in oil (13–24%), and tomato seed oil was dominated by linoleic acid (38–73%), with a sterol content of 1.55–12.3 mg/g and a tocopherol content of 0.94–1.11 mg/g, a lipid profile comparable to that of vegetable oils, which are highly nutritionally valuable. The analysis of the separation processes indicated that wet methods achieved a purity of 90–97% but with losses of micronutrients and high water consumption, while dry separation methods offered efficiencies of 68–70% but were more easily scalable in the industry. Based on these data, the authors attested that processing the entire TP, without prior separation, may represent a more efficient and sustainable alternative. This conclusion is in line with the integrated biorefining approach described by Almeida et al. [16], which highlights the advantages of simultaneously valorizing polar and lipophilic fractions. From biological and clinical points of view, lycopene derived from TP is associated with antioxidant and cardioprotective effects, with benefits reported at doses of 5–7 mg/day for the prevention and reductions in LDL cholesterol of approximately 14% when administered at ~60 mg/day for 3 months.

Kopjar et al. [Contribution 9] investigated the effect of adding disaccharides (sucrose and trehalose) on the encapsulation of polyphenols from chokeberry juice onto rice and pea protein matrices. They used freeze-drying to obtain several functional protein aggregates for various food applications. The complexation of the ingredients was achieved by two technological strategies, namely, the simultaneous complexation of all components and sequential complexation, in which the proteins were first associated with disaccharides and subsequently with chokeberry juice. The results demonstrated that the type of protein, the type of disaccharide, and the complexation method significantly influenced the efficiency of polyphenol adsorption. In the case of rice proteins, the aggregates without disaccharides had the highest contents of total polyphenols (28.03 mg GAE/g) and proanthocyanidins (8.57 mg PB2E/g), while the addition of sucrose or trehalose caused a reduction in these to 22.41–24.04 mg GAE/g and 6.36–7.28 mg PB2E/g, respectively. In contrast, for pea proteins, disaccharides had a favorable effect, with the content of total polyphenols increasing from 21.25 mg GAE/g in the aggregates without disaccharides to 21.42–26.44 mg GAE/g and proanthocyanidins from 5.56 mg PB2E/g to 6.37–9.45 mg PB2E/g, values comparable to those previously reported by Roopchand et al. [17] for the adsorption of berry polyphenols on globulin-rich vegetable protein matrices. The chromatographic analyses revealed the efficient encapsulation of the main anthocyanins (cyanidin-3-galactoside, cyanidin-3-arabinoside, and cyanidin-3-glucoside), as well as chlorogenic and neochlorogenic acids. The antioxidant assays (DPPH, ABTS, FRAP and CUPRAC) indicated strong correlations between polyphenol/proanthocyanidin content and antioxidant activity (r = 0.89–0.99). The FTIR-ATR recordings confirmed the formation of aggregates through non-covalent interactions, predominantly hydrogen bonds and hydrophobic interactions, evidenced by changes in the amide bands I–III, results in agreement with the mechanisms described by Le Bourvellec and Renard [18] for protein–polyphenol complexes.

## Data Availability

Data sharing is not applicable.
